# The Moderating Role of Corporate Social Responsibility in the Association of Internal Corporate Governance and Profitability; Evidence from Pakistan

**DOI:** 10.3390/ijerph18115830

**Published:** 2021-05-28

**Authors:** Jihai Lu, Sohail Ahmad Javeed, Rashid Latief, Tao Jiang, Tze San Ong

**Affiliations:** 1 College of Education, Zhejiang University, Hangzhou 310058, China; mreastlu@163.com; 2 Hangzhou College of Commerce, Zhejiang Gongshang University, Hangzhou 311500, China; jtao@263.net; 3 School of Finance and Economics, Jiangsu University, Zhenjiang 212013, China; rashidlatief@ujs.edu.cn; 4 School of Business and Economics, Universiti Putra Malaysia, Serdang 43400, Malaysia; tzesan@upm.edu.my

**Keywords:** corporate social responsibility (CSR), internal corporate governance, firm performance, manufacturing firms

## Abstract

At present, climate and other environmental problems are arising because of the development of the industrial sector at a large level. The industrial sector is supposed to be a major cause of climate change problems that lead to global warming. Therefore, corporate social responsibility (CSR) with the help of corporate governance is an imperative approach to control these social problems. Consequently, in the context of the organizational and management theory, agency theory, and the stakeholder theory, this study focuses on important factors of internal corporate governance such as chief executive officer (CEO) power, the board size, independence, ownership concentration, managerial ownership, and audit quality for improving the profitability of firms. Moreover, this study considers corporate social responsibility as a controlling and moderating factor for firm performance and internal corporate governance. We employed ordinary least square (OLS) for endogeneity testing, fixed effect (FE), generalized method of moments (GMM), and feasible generalized least square (FGLS) on data of Pakistani firms for the period of 2010–2019. The results of this study demonstrate the following outcomes: firstly, all internal corporate governance factors are positively linked with firm performance; secondly, corporate social responsibility (CSR) is the most valuable tool for improving profitability. Importantly, this study suggests that all internal corporate governance factors are positively linked with firm performance because of the interactive role of corporate social responsibility (CSR). This study practically contributes to the literature by suggesting the imperative role of corporate social responsibility (CSR) for internal corporate governance, which may help to reduce climate and social problems.

## 1. Introduction

At present, the world is suffering severe environmental glitches and the problem getting worse over time; therefore, institutions and governments are working to minimize or remove these environmental problems [1]. Previous researchers reported that the industrial sector is highly responsible for these environmental and social problems, especially the firms of developing economies [2]. Therefore, corporate social responsibility (CSR) is measured as a major tool to motivate insider corporate governance for social actions [3]. CSR practices normally engaged firm-level governance to participate in social and environmental activities [4]. The internal corporate governance (ICG) is an imperative body to control and monitor corporate social practices. Generally, profit maximization is a major motive of every business or firm. Therefore, corporate governance adopts various techniques to increase the firm profit. The majority of studies revealed that inside corporate governance is highly important for firm profitability and other actions [5,6].

On the other hand, few researchers disclosed the negative or insignificant connection of corporate governance with firm profitability [7,8]. However, no prior study specifically described the reason for this positive, negative, or insignificant affiliation between corporate governance and profitability. Before this, most studies inspected only a single or few dimensions of internal corporate governance. Therefore, this study considers various internal corporate governance elements. Additionally, this study proposes that CSR investigates the reason for this positive, negative, or insignificant relationship, because corporate social responsibility is supposed to be an effective tool for internal corporate governance and has a positive relationship [9]. Besides, Wu, et al. [10] stated that information disclosure serves as an important tool to control the information asymmetry between internal corporate governance and other stakeholders. CSR enhances the firm reputation and, therefore, corporate governance may involve social practices [11].

However, this study has discovered various corporate governance factors according to the guidelines of the Securities and Exchange Commission of Pakistan (SECP) [12]. For instance, the chief executive officer (CEO) and supervisory board, which consists of board independence and size. Moreover, we use an ownership structure that consists of ownership concentration and managerial ownership because it plays an imperative part in internal corporate governance. Similarly, audit quality is also a very important factor of internal corporate governance. For firm performance, this study uses economic value added (EVA) and sustainable growth rate (SGR). To support this study, firstly, the organization and management theory supported the role of CEO power [13]. Secondly, the agency theory supported board structure, ownership structure, and audit committee [14]. Thirdly, the stakeholder theory supported the role of CSR as moderating factor for the link between firm performance and internal corporate governance [15].

This study applied multiple statistical methods for empirical investigation. Firstly, we employed ordinary least squares (OLS) to discover the endogeneity. Secondly, we employed the fixed effect technique to overcome the unobservable heterogeneity. Thirdly, we employed the generalized method of moments (GMM) for correcting the endogeneity issues from our data. Lastly, for the robustness test, feasible generalized least square (FGLS) has been employed to overcome the overall heterogeneity. Our results reported that internal corporate governance factors are positively linked with firm performance. Secondly, our results discovered that CSR is positively connected with firm performance. Importantly, this study highlights that CSR is the highly valuable moderating factor for the positive connection between internal corporate governance factors and firm performance.

The outcomes of our study have various suggestions for policymakers, shareholders, owners, investors, institutes, and governments. This study recommended the role of corporate social responsibility to control and monitor internal corporate governance. Thus the Government of Pakistan (GOP) must tighten these regulations for controlling internal corporate governance and enhancement of profitability. Additionally, this study provides directions for GOP and other institutes for developing corporate social practices. The conclusion of this study is highly valuable for the firms of developing and developed economies to control and monitor the internal corporate governance factors for better outcomes.

**Figure 1:**
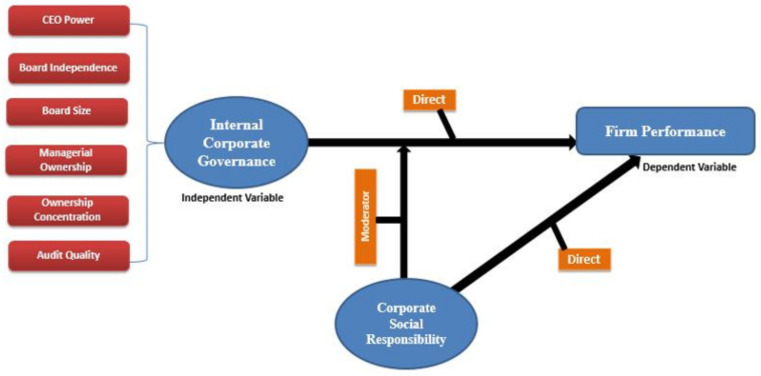
Conceptual Roadmap. CEO: chief executive officer.

The rest of the sections of this paper are categorized into the following parts. Section 2 explains the theoretical and literature framework for hypothesis development. Section 3 reveals the sample selection, data collection, contextual analysis of social and corporate governance practices in Pakistan, variables’ construction, and empirical methods. Section 4 sheds light on the empirical findings with a discussion. The last section reveals the conclusion and policy implications. Figure 1 represents the conceptual framework of the study.

## 2. Theoretical Analysis

Theoretically, various theories shed light on the imperative role of the internal corporate governance factors for firm performance. CEO power as compensation has a significant role in firm performance. In this context, firstly, the organization and management theory stated that a powerful CEO may properly implement their decisions [13]. This theory sheds light on the imperative of the powerful CEO to reduce the managerial unnecessary expenses. A powerful CEO controls and monitors investment decisions that minimize the chances of managerial personal use. Therefore, this theory encouraged firms to have a powerful and strong CEO for the improvement of firm performance.

Furthermore, the firm board has an imperative role in firm performance. In this support, the agency theory exposed that board members endow with monitoring and organizing function on managers actions [14]. Agency theory supported the role of board size in firm performance. The involvement of all board members may create fairness in decision making that builds the firm trust level and attracts more investors. Besides, agency theory also sheds light on the auditing practices for reducing agency costs. The audit committee is a valuable approach to minimize the information asymmetry between owners and management because an independent audit committee focuses on the organization’s transparent actions that build confidence between owners and managers [16]. Generally, the audit committee is accountable for making independent audits and monitors both internal and external auditors for transparency. The agency theory also reveals the imperative role of dividend policy in firm performance. This theory focuses on the difference of opinion between principals and agents [14]. The connection between the agent and principal is intrinsically covered with, firstly, the information asymmetry that exists between principal and agent, more on the dispute of interest between them [17]. The timely dividend payment discourages managers from overinvesting and enhances shareholders’ confidence.

Thirdly, stakeholder theory supported the role of corporate social responsibility for firm performance [15]. Internal corporate governance always looks for means to enhance firm profit and, therefore, they considered CSR to improve the firm image, which automatically enhances the firm profit [18]. Stakeholder theory also supported the moderating role of CSR because internal corporate governance uses effective ways for saving the interest of the firm stakeholders with the help of CSR [9,19]. CSR puts pressure on the firm internal corporate governance to work for firm performance and a positive firm image in the market. The concern of stakeholders is highly important for corporate governance practices and CSR practices assure all stakeholders [20].

## 3. Literature Review and Hypothesis Development

### 3.1. Internal Corporate Governance Factors and Firm Performance

This study divided internal corporate governance into five categories by following the securities and exchange commission of Pakistan [12]. Firstly, this study considers senior executives or top executives as the major decision bodies in the firm. The senior executives consist of the chief executive officer (CEO) power as compensation. Secondly, this study considers board structure or composition, which includes board members, board independence, and remuneration committee. Thirdly, this study considers the structure of ownership, which is further classified as managerial and concentrated ownership. Fourthly, this study focuses on auditing practices as internal corporate governance.

Berger, et al. [21] reported firms’ innovational activities normally depend upon the CEO’s decision-making. Papadakis [22] reported that shareholders consider the CEO as a leader to enhance firm value. Coles, et al. [23] investigated the industrial tournament incentives as a CEO power with financial elements of investment, value, policies, and risk. They highlighted that a powerful CEO is highly valuable for the above financial elements. Moreover, the CEO is assumed as the most powerful in developing countries such as Pakistan, where this position is mainly held by the family member [24]. The CEO is generally supposed to the most powerful member of the organization [25]. Most of the prior literature supported the role of the CEO for firm financial performance. Moreover, Daily and Johnson [25] also found that a powerful CEO not only works for the enhancement of profit, but also for the long-run survival of the organization. In addition, they have measured CEO power in four different ways and reported that a powerful CEO in all terms is valuable for the improvement of firm profit. Furthermore, Garcia-Sanchez, et al. [26] have investigated the connection of CEO power with integrated reporting. After applying logistic regression on 10,819 observations, they demonstrated that a powerful CEO is valuable for transparency, which leads to creation of firm value. In this aspect, Busco, et al. [27] also supported the role of a powerful CEO for information disclosure. Muttakin, et al. [28] concluded that CEO power is highly valuable for information disclosure, which leads to higher profit.

Besides, board composition is an imperative internal corporate governance factor for firm performance. Raheja and analysis [29] emphasized that board composition and size are functions of the firm major decisions making, and automatically affect the firm performance. Both of these factors of internal corporate governance have much importance for firm performance [30]. Previous scholars believe that expert and adept members on a board can play a vital role in the enhancement of firm performance [31]. Ansari, et al. [32] selected the Pakistani automobile sector to explore the link between firm performance and board size. Their results also suggested positive connections. A study conducted in Hong Kong reported that board independence plays a positive part in the improvement of profitability [33].

Thirdly, this study considers ownership structure as internal corporate governance, which includes ownership concentration and managerial ownership [24]. Generally, ownership structure involves policy-making for the improvement of industrial activities. The Pakistani markets are extremely concentrated owing to family ownership. Abbas, et al. [34] stated that ownership concentration in Pakistan has a significant role in enhancing firm performance. Besides, Yasser and Mamun [35] also inspected the connection between concentrated ownership and firm performance in Pakistan. They discovered a positive relationship. Similarly, multiple studies provided evidence of positivity about the association between firm performance and managerial ownership [24,36]. Kapopoulos and Lazaretou [37] stated that ownership structure is entirely important for the improvement of firm performance. Demsetz and economics [38] also supported the role of large shareholders in firm performance because large shareholders are always interested in the long-run profitability of the firm. Moreover, Raimo, et al. [39] suggested that integrated reporting is an interesting tool for firm short-, medium-, and long-run survival. Thus, after the probe of 152 international firms, they reported that institutional ownership is imperative for integrated reporting, while managerial ownership, state, and concentrated ownership are negatively linked with integrated reporting.

Fourthly, this study focus on auditing practices as an internal corporate governance factor. According to Waweru [40], the audit is an internal part of firm corporate governance. Furthermore, Masood, et al. [41] exposed that audit is an internal governance tool to improve and correct the firm financial affairs, which have huge importance for government and private industries. Matoke, et al. [42] probed the impact of audit quality on firm performance. They established that audit quality is helpful to improve firm performance. Besides, Al Ani, et al. [43] and Sattar, et al. [44] also supported the role of auditing practices for the improvement of firm performance. By following theoretical and empirical analysis, this study has developed various hypotheses:

**Hypothesis 1**:  *Top executives have a positive association with firm performance*.

**Hypothesis 2**:  *Board composition has a positive association with firm performance*.

**Hypothesis 3**:  *Ownership structure has a positive association with firm performance*.

**Hypothesis 4**:  *Audit quality has a positive association with firm performance*.

### 3.2. Corporate Social Responsibility (CSR) and Firm Performance

In recent years, there has been huge debate related to CSR owing to the higher demands from shareholders and communities. Shareholders and societies considered firms’ irresponsible social actions as costly [45]. Moreover, the trust level between shareholders and management is poor in emerging markets; therefore, this external control can improve this trust level. This external verification control works as a strong external corporate governance mechanism [46]. There is mixed evidence available for the association between CSR and firm performance, as some studies reveal positive effects, some reveal negative effects, and some reveal no effect [47,48,49].

Such uncertain outcomes generate a research space, permitting various scholars to probe the actual and satisfying outcomes. There is a proper theoretical foundation available that suggests the importance of CSR for firm performance. Similarly, Oeyono, et al. [50] stated that firms, by having social activities, can satisfy communities and enhance the relationship with other stakeholders. Thus, CSR is assumed as a strong external corporate governance factor for firm performance. Furthermore, Aerts, et al. [51] also found that firms with CSR actions have better relations with their stakeholders, which lead to higher profit. Besides, multiple studies proved that CSR is a key factor to improve relations with societies and other stakeholders, which automatically lead to higher profitability [24].

Kong, et al. [52] have reviewed various past published research papers on corporate social responsibility and business outcomes. They finally concluded that corporate social practices are valuable for positive business outcomes. Naseem, et al. [53] also highlighted the corporate social practices for business performance in the context of Asia. Their outcomes show that business performance is enhanced when a firm participates in corporate social practices because it wins the shareholders’ confidence. Hence, this study has developed the following hypothesis:

**Hypothesis 5**:  *Corporate social responsibility (CSR) has a positive linkage with firm performance*.

### 3.3. The Moderating Role of CSR

This study used CSR as an imperative tool to control and monitor the internal corporate governance practices for the maximization of profit [9]. Internal corporate governance is always interested in investing in CSR actions for the improvement of firm reputation in the market [54]. Besides, they reported that a strong corporate governance mechanism can overcome the conflicts between agents and owners by using the actual meaning of CSR practices. Board monitoring is a beneficial tool to control and monitor corporate over-investing [55]. Moreover, stakeholder theory stated that a firm does not only have the sole purpose to earn a profit, but also to satisfy its shareholders [15]. Shareholders consider those firms that engage in social practices as more responsible. This practice enhances the long-term value of a firm.

Internal corporate governance always looking for means to enhance the firm profit and therefore they considered CSR to improve the firm image which automatically enhances the firm profit [56]. Besides, Khan, et al. [57] stated that the majority of the internal corporate governance elements are positively connected with CSR in an emerging economy. Jamali, et al. [58] reported that CSR serves as an important controlling mechanism to motivate the corporate governance factors for participation in social practices. Said, et al. [59] exposed that corporate governance as the audit committee and Government ownership has positive effects on the corporate social practices in Malaysia. Furthermore, Zhuang, et al. [60] highlighted that CSR practices automatically enhance the worth of board composition by disclosing the information about board members. Board size and independence significantly improve the CSR practices and firm performance [61]. 

Besides, Lone, et al. [62] proposed that corporate governance practices are entirely important for CSR activities in Pakistan. Moreover, internal corporate governance has impressive results related to CSR practices in Pakistan [63]. Majeed, et al. [64] investigated the internal corporate governance elements as, board member independence, the board size, ownership structure, and firm size related to the CSR activities in Pakistan. Their finding supported the role of internal corporate governance for CSR practices as having a positive relationship between them. Gul, et al. [65] also demonstrated that CSR practices play an imperative role to control and motivate the internal corporate governance elements. Corporate governance internal characteristics are very important for CSR practices in Pakistan and enhance shareholders’ wealth [66]. Furthermore, Javeed and Lefen [24] found that CSR and firm performance have a positive connection in Pakistan. They majorly concluded that internal corporate governance as CEO power, concentrated and managerial ownership are the key factors to improve the corporate social practices which directly enhances firm profit as well. 

Cong and Freedman [67] discovered that corporate governance has a positive connection with environmental disclosure practices. Besides, Li, et al. [68] explained that a powerful CEO is beneficial for environmental practices and firm profitability. They concluded that environmental disclosure is positively associated with the firm internal governance factor CEO. CEO considered social practices as an opportunity for earning management [69]. Kock, et al. [70] explained that environmental performance is entirely associated with internal corporate governance as, the board of directors, managerial incentives, auditor, and corporate control. Environmental regulations push corporate governance to perform social action for the sake of society in Pakistan [71]. Major element of corporate governance-CEO can improve firm sustainable and environmental practices. 

Environmental regulations force the CEO to adopt environmental practices for the improvement of the firm image and which leads to higher profit [72]. Javeed and Lefen [24] concluded that corporate governance internal elements CEO, ownership concentration, and managerial ownership have an advantageous role in the implementation of corporate social practices in Pakistan. They further reported that these social practices are also valuable for firm performance. Corporate social activities are generally performed by good governance [73]. A better corporate governance system is always interested to perform environmental and social practices for the sake of a better image in the eyes of stakeholders which automatically enhances profitability [74].

On the other hand, various studies showed that board characteristics are also valuable for corporate environmental practices and disclosure [75,76]. Besides, Rabi [77] inspected the connection between board characteristics (board size, ownership, size) and environmental disclosure in Jordan. They found that all these board characteristics and environmental disclosure are positively linked. Moreover, Uwuigbe [78] highlighted that firms with managerial ownership are more willing to participate in social practices. Similarly, ownership concentration and board independence are important tools to enhance the firm disclosure practices which automatically forces firms to participate in social and environmental actions [79]. 

Ownership concentration have a positive association with social practices [80]. Besides, corporate social practices are very important for auditing because auditing reveals that what firms spending on environmental practices [81]. There is mixed literature available which supported that CSR serves as an external control for internal corporate governance for example CSR and CEO power, concentrated and managerial ownership [24], CSR and board independence, size [82,83], and CSR and audit quality [84]. This study has made the following hypothesis:

**Hypothesis 6**:  *Corporate social responsibility (CSR) positively influences the association between internal corporate governance factors and firm performance*.

Here, internal corporate governance includes all factors such as CEO power, board members, board independence, managerial ownership, ownership concentration, and audit committee.

## 4. Research Approach

### 4.1. Description of the Sample

We employed secondary data (panel data) as choosing Pakistani firms. This article used various firms from various sub-sectors, for example, the textile sector which consists of weaving, woolen, spinning, and composite. Specifically, the chemical, cement, oil and gas exploration, fertilizer, pharmaceutical, oil and gas marketing, synthetic and rayon, refinery, engineering, automobile parts and accessories, automobile assembler, transport, glass and ceramics, cable and electrical goods, leather and tanneries, food and personal care products, technology and communication, paper and board, sugar and allied industries power generation and distribution have been used to complete this study.

These sectors include a total of 475 firms which covers 87% of the Pakistan Stock market. This study selected a sample on multiple objectives firstly firms that disclose social practices in annual reports are part of this probe. Secondly, firms with data missing or declared defaulter by the Pakistan Stock Exchange (PSX) are excluded from the sample [24,85]. Therefore, 136 defaulting and non-disclosed data firms dropped from the sample. Finally, this study used 339 firms for this probe. There are multiple sources used to gather the secondary data reported by [24,71] such as the State Bank of Pakistan (SBP), the Pakistan Stock Exchange (PSX), the SECP, sustainability, and annual reports of the firms for 2010–2019. 

### 4.2. CSR and Corporate Governance Practices in Pakistan

The corporate governance connection with firm performance is extremely important for developing economies. Corporate governance plays an imperative role for firm performance which leads to the economic development of the states. The code of corporate governance in Pakistan was established in 2002, and this code extended with further important amendments in 2012 [86]. Therefore, the corporate governance idea in Pakistan has not long passed history because it was developed just one decade ago. Normally, firms of developing countries like Pakistan, India, and Bangladesh are not much involved in the improvement of corporate governance activities. These countries strictly bear economic problems, safety, and health issues at the workplace, low level of environmental and employee safety, violation of human rights in the form of child labor [24]. 

Moreover, Pakistan is a country that is suffering from various issues such as unbalanced political and economic conditions, health environment, moreover industrial and energy disasters, communal divergence, extensive corruption, and an inadequate controlling outline [71]. On a big platform, overall these issues have repercussions for the industries. Generally, Pakistan’s industrial structure has the majority of family ownership. According to Cheema and Din [87], 60% of firms in Pakistan are controlled by family ownership, and only 40% are controlled by other ownership. In addition to this, according to Gamerschlag, et al. [88], corporate governance activities are not much developed in Pakistan. Concerning this, the SECP is highly committed to implement better corporate governance and social practices for improved performance [89]. There is a lack of work for corporate governance and its other factors in developing countries [90]. Researchers believed that better corporate governance is an alternative model to improve the firm performance and economic conditions of the country. Pakistan Stock Exchange is an emerging market [91]. 

Despite facing all these issues, Pakistan is a law-abiding country that sturdily focuses on the protection and safety of shareholders the development of the stock market [71]. Pakistan has important capital and financial market have numerous social and political practices as a comparison to other developed economies [92]. Additionally, the world is facing global warming threats and scholars believed that the industrial sector is the major cause of environmental problems [93]. Therefore, this study presents corporate social responsibility’s importance in corporate governance for improving profitability and reducing environmental problems. Focusing on the above circumstance, there is an intense need to explore corporate governance and social activities in Pakistan. 

### 4.3. Variable Measurement

Table 1 reveals the measurements of the variables.

### 4.4. Analysis Techniques

#### 4.4.1. Panel Data Issues

Prior studies revealed that panel data normally carries endogeneity issues. While using panel data for empirical evaluation, the endogeneity issue normally occurs because error terms correlate with explanatory variables, which produces unreliable and biased outcomes [71]. Similarly, econometrics exposed that endogeneity issues happen with the connection between error term and explanatory variables. Nonetheless, these issues available for every field like economics, finance, etc. For example, endogeneity issue highlighted in few prior studies about corporate governance [95], compensation of executives [98], board composition and managerial ownership [99,100], firm control [101], and financial and investment policies [102]. However, most researchers have not mentioned the endogeneity concerns in their studies as Antonakis, et al. [103] and Hamilton and Nickerson [104] exposed that 90 percent of published articles did not highlight the endogeneity concern.

Besides, panel data also carries heteroskedasticity issue, which normally occurs when the variables’ standard errors have observation over a particular amount of time, which is not constant [105]. Heteroskedasticity generally occurs in two circumstances, conditional and unconditional situations. The conditional situation is explained as the variable volatility available which leads to the future time of low and high volatility which cannot be recognized. Unconditional situation refers to a position where the future time of low and high volatility can be recognized.

#### 4.4.2. Solution for Panel Data Issues

Moreover, Li [106] presented numerous procedures to overcome the endogeneity issues from panel data. For example, the third-factor effect, instrumental variable method, and the lagged dependent variable can overcome endogeneity such as the effect of control variables. But, his findings highly suggested the use of the GMM model for covering endogeneity. Additionally, the fixed-effect model is considered the best approach for the unobservable time-invariant. After the evaluation of all methods majority of the scholars reported that 2SLS and GMM methods are the most impressive and significant approaches to deal with and control the endogeneity from panel data [68,106,107,108,109,110,111].

This study used firm-level panel data, therefore, it is a need to control the heteroskedasticity and autocorrelation problem. According to Wooldridge [112] and Baltagi [113] firstly conducted the test for serial autocorrelation between the residuals over the specified period with the use of the FGLS model. Moreover, they suggested that the FGLS model can robust the autocorrelation to evaluate the parameter in the equation. Greene [114] exposed that the FGLS model is best to reduce the possible heteroskedasticity from the panel and cross-sectional data. Furthermore, multiple studies recommended that autocorrelation and heteroskedasticity problems can be overcome with the use of FGLS [113,115,116,117].

### 4.5. Empirical Estimation Procedure

Generally, OLS regression is a valuable technique to find the relationship between dependent and independent variables. Thus, we firstly employed OLS regression for each equation to identify the endogeneity by using the Durbin-Wu-Hausman experiment [111,118,119]. Secondly, a fixed-effect model has been employed to cover the inaudible heterogeneity based on the Hausman test [111,119]. The Hausman results permitted us to employ a fixed-effect model instead of a random effect model. 

Thirdly, for more accuracy, this study has been used the generalized method of moments (GMM) for solving the endogeneity issues. Prior researchers strongly believed that the use of GMM is the most suitable approach for correcting endogeneity compared to other methods [71,109,110]. Finally, this study employed the FGLS model as a robustness test based on the Hausman test to investigate, the heteroscedasticity and autocorrelation from panel data [113,115,116,117].

#### 4.5.1. Model Construction

##### ICG and Firm Performance

Various scholars have developed the econometric equation for the investigation of internal corporate governance association with firm performance [120,121,122,123]. Similarly, we have developed the following equation for internal corporate governance and firm performance.

(1) $$Y_{i,t}=\alpha_{1}+\beta_{1}X_{1i,t}+\beta_{2}X_{2i,t}+\beta_{3}X_{3i,t}+\beta_{4}X_{4i,t}+\beta_{5}X_{5i,t}+\beta_{6}X_{6i,t}+\gamma_{1}Z_{i,t}+\mu_{i,t}$$

In the Equation (1), $Y_{i,t}$: the firm performance (EVA, SGR) of firms *i* at year *t*, creating two sub equations for each index; chief executive officer (CEO) power; $X_{1i,t}$: board independence (BI); $X_{2i,t}$: board size (BS); $X_{3i,t}$ managerial ownership (MO); *X*_4*i,t*_: ownership concentration (OC); *X*_5*i,t*_: audit quality (AQ); *X*_6*i,t*_: control variables of firm *i* at year *t*; $\mu_{i,t}$: error term; $\alpha_{n}$: constant term, *n* = 1; $\beta_{m},\;\gamma_{n}$: coefficients to be estimated; *m* = 1, 2, 3, 4, 5, 6.

##### CSR and Firm Performance

Multiple researchers have formed the econometric equation for the evaluation between corporate social responsibility and firm performance, such as [124,125]. Therefore, we have developed the following econometric equation:

(2) $$Y_{i,t}=\alpha_{1}+\beta_{1}X_{1i,t}+\gamma_{1}Z_{i,t}+\mu_{i,t}$$

In the Equation (2), $Y_{i,t}$: the firm performance (EVA, SGR) of firms *i* at year *t*, creating two sub equations for each index; $X_{1i,t}$: corporate social responsibility (CSR): control variables of firm *i* at year *t*; $\mu_{i,t}$: error term; $\alpha_{n}$: constant term, *n* = 1; $\beta_{m},\;\gamma_{n}$: coefficients to be estimated; *m* = 1.

##### The Moderating Role of CSR

This study used various internal corporate governance factors. To find the relationship between internal corporate governance and firm performance, this study applies corporate social responsibility as a moderating factor. Therefore, this study developed the following equation:

(3) $$Y_{i,t}=\alpha_{1}+\beta_{1}X_{1i,t}+\beta_{2}X_{2i,t}+\beta_{3}X_{1i,t}\times X_{2i,t}+\gamma_{1}Z_{i,t}+\mu_{i,t}$$

In the Equation (3), $Y_{i,t}$: the firm performance (EVA, SGR) of firms *i* at year *t*, creating two sub equations for each index; $X_{1i,t}$: internal corporate governance (ICG_af_); $X_{2i,t}$: corporate social responsibility (CSR); $X_{1i,t}\times X_{2i,t}$: the interaction between internal corporate governance factors and CSR of firm *i* at year *t*; $Z_{i,t};$ control variables of firm *i* at year *t*; $\mu_{i,t}$: error term; $\alpha_{n}$: constant term, *n* = 1; $\beta_{m},\;\gamma_{n}$: coefficients to be estimated; *m* = 1, 2, 3. In this equation, internal corporate governance (ICG_af_) represents all internal factors such as CEO power, board independence, board size, managerial ownership, ownership concentration, and audit quality.

## 5. Results and Discussion

### 5.1. Results

This study conducted descriptive statistics and correlation tests before testing the hypothesis. Table 2 reveals the mean and standard deviation of all independent and dependent variables. A total of 3950 observations were used for 10 years’ firm-level data. Moreover, Table 2 also shows the correlations of all variables.

Afterwards, this study conducted an endogeneity test because panel data has been used to probe the connection between internal corporate governance and firm performance with the moderating role of CSR. Thus, panel data normally caries endogeneity issues for empirical studies [71,126]. Therefore, we applied OLS regression to conduct the test for the detection of endogeneity in our panel data.

The use of OLS regression normally occurred to find the upshot of independent variables on the dependent variables. Our main equations have two dependent variables such as EVA, and SGR, but we used each independent variables (CEO POWER, BS, BI, OC, MO, AQ, and CSR) as dependent variable one by one to find the endogeneity bias (Beiner et al. (2006); Schultz et al. (2010); and Wintoki et al. (2012)). 

The Durbin–Wu–Hausman test is conducted using OLS regression and the results are explained in the Table 3. The significant values of F statistics reveal that our independent variables are endogenous. Furthermore, these results show that there is a correlation between residuals and independent variables. Thus, if there are endogenous variables in the model then there is a need to employ an appropriate techniques to cover the endogeneity issues [120]. Table 3 presents the endogeneity test for internal corporate governance variables.

Finally, Table 4 shows the outcomes of the link between all internal corporate governance variables and firm performance. For instance, model 1 highlights the results of all internal corporate governance variables CEO POWER, BI, BS, MO, OC, and AQ with EVA using FE (β = 0.004, *p* = 0.01, β = 0.015, *p* = 0.01, β = 0.008, *p* = 0.01, β = 0.006, *p* = 0.01, β = 0.012, *p* = 0.01, β = 0.240, *p* = 0.01). Model 2 highlights the results of all internal corporate governance variables CEO POWER, BI, BS, MO, OC, and AQ with EVA using GMM (β = 0.005, *p* = 0.01, β = 0.018, *p* = 0.01, β = 0.004, *p* = 0.01 β = 0.005, *p* = 0.01 β = 0.009, *p* = 0.01, β = 0.237, *p* = 0.01). The Hausman test value supported the employment of fixed effect model, as (β = 58.56, *p* = 0.01).

Furthermore, Table 4 explains model 2, which reveals the outcomes of all internal corporate governance variables CEO POWER, BI, BS, MO, OC, and AQ with SGR using FE such as (β = 0.001, *p* = 0.01, β = 0.003, *p* = 0.01, β = 0.002, *p* = 0.01, β = 0.001, *p* = 0.01, β = 0.003, *p* = 0.01, β = 0.060, *p* = 0.01). Model 4 reveals the outcomes of all internal corporate governance variables CEO POWER, BI, BS, MO, OC, and AQ with SGR using GMM such as (β = 0.011, *p* = 0.01, β = 0.004, *p* = 0.01, β = 0.001, *p* = 0.01, β = 0.002, *p* = 0.01, β = 0.003, *p* = 0.01, β = 0.059, *p* = 0.01). Similarly, the Hausman test value also supported the employment of fixed effect model as (β = 56.87, *p* = 0.01). Hence, our results proved that all internal corporate governance variables are positively linked with firm performance.

Moreover, Table 5 displays the outcomes of the link between all CSR variables and firm performance. Model 3 reveals the outcomes of CSR with EVA using FE and GMM, respectively, such as (β = 0.155, *p* = 0.01 and β = 0.144, *p* = 0.01). The Hausman test value confirmed the employment of the fixed-effect model as (β = 83.86, *p* = 0.01). Model 4 highlights the outcomes of CSR with SGR using FE and GMM, respectively, such as (β = 0.038, *p* = 0.01 and β = 0.035, *p* = 0.01). Likewise, the Hausman test value confirmed the employment of the fixed-effect model as (β = 13.54, *p* = 0.01). Our results stated that CSR and firm performance are positively linked.

Lastly, Table 6 shows the results of the moderating role of CSR on the link between internal corporate governance variables and firm performance. Model 5 reveals the outcomes of all internal corporate governance variables with the interaction of CSR such as CEOPOWER × CSR, BI × CSR, BS × CSR, MO × CSR, OC × CSR, and AQ × CSR with EVA using FE such as (β = 0.001, *p* = 0.01, β = 0.471, *p* = 0.01, β = 0.856, *p* = 0.01, β = 2.153, *p* = 0.01, β = 1.935, *p* = 0.01, β = 10.88, *p* = 0.01). Model 5 also reveals the outcomes of all internal corporate governance variables with the interaction of CSR such as CEOPOWER × CSR, BI × CSR, BS × CSR, MO × CSR, OC × CSR, and AQ × CSR with EVA using GMM such as (β = 0.002, *p* = 0.01, β = 0.524, *p* = 0.01, β = 0.875, *p* = 0.01, β = 1.976, *p* = 0.01, β = 1.760, *p* = 0.01, β = 11.87, *p* = 0.01). The Hausman test value allowed to use of fixed effect model as (β = 44.03, *p* = 0.01). In addition, model 6 reveals the outcomes of all internal corporate governance variables with the interaction of CSR such as CEOPOWER × CSR, BI × CSR, BS × CSR, MO × CSR, OC × CSR, and AQ × CSR with SGR using FE such as (β = 0.009, *p* = 0.01, β = 0.117, *p* = 0.01, β = 0.214, *p* = 0.01, β = 0.538, *p* = 0.01, β = 0.483, *p* = 0.01, β = 2.722, *p* = 0.01). Model 6 also reveals the outcomes of all internal corporate governance variables with the interaction of CSR such as CEOPOWER × CSR, BI × CSR, BS × CSR, MO × CSR, OC × CSR, and AQ × CSR with SGR using GMM such as (β = 0.002, *p* = 0.01, β = 0.131, *p* = 0.01, β = 0.218, *p* = 0.01, β = 0.494, *p* = 0.01, β = 0.440, *p* = 0.01, β= 2.968, *p* = 0.01). Likewise, the Hausman test value allowed to use of fixed effect model as (β = 45.44, *p* = 0.01). Finally, our results supported our hypothesis, which reveals that all internal corporate governance variables are positively linked with firm performance with the moderating role of CSR.

### 5.2. Additional Test

This study applied the fixed feasible generalized least square (FGLS) as an additional test for further robustness of the results. Table 7 reveals the results of ICG association with FP, CSR with FP, and the association between ICG and FP with the moderating role of CSR as a robustness test. These robustness results with the FGLS model confirmed the results of the previous models. Furthermore, these robustness results support the previous findings.

### 5.3. Discussion

The results of relationship between ICG (CEO POWER, BI, BS, MO, OC, and AQ) and firm performance (EVA and SGR) showed various outcomes after employing fixed effect, GMM, and FGLS models. Firstly, our results confirmed that a powerful CEO in the term of compensations have a significant and positive role for firm performance. Javeed and Lefen [24] also supported that a powerful CEO is highly beneficial for firm performance in Pakistan. Various other studies also confirmed these results [121,122,123]. Theoretically, the organizational and management theory provided evidence for the positive relationship between CEO Power and firm performance [124]. 

Secondly, our findings exposed that BI and BS have a positive linkage with internal and external firm performance measures. The board structure has a significant role in firm performance in Pakistan [120]. Multiple scholars supported this evidence [127,128]. Independent board can make decisions for good governance without the pressure of owners which leads to higher profitability. Besides, various previous studies provided evidence to support the connection of BS and firm performance [129,130,131,132]. Pearce and Zahra [133] exposed that adept members on the board are imperative for firm performance. In addition to this, agency theory supported this positive connection, because agency cost can be reduced with expert board members [14].

Thirdly, our results presented that MO and OC are positively linked with internal and external firm performance. To support these outcomes, Javeed and Lefen [24] discovered that ownership structure and firm performance in the Pakistani market have a positive connection. Additionally, these findings are similar to many previous scholars such as [133,134,135]. Kim, et al. [136] found that MO is an imperative means for improving firm performance in developing countries. In addition to this, to support the association between OC and firm performance different researchers provided evidence [137,138,139]. Furthermore, agency theory also supported that ownership structure can minimize the agency cost which may gain higher profit [14]. Pakistani market has higher family ownership and therefore managers and other shareholder’s involvement in the ownership may reduce agency conflict. These practices enhance international shareholder’s confidence as well. 

Fourthly, the findings of this study reported that AQ and firm performance have a positive connection. In this context, Sattar, Javeed and Latief [44] discovered that the quality of audit is highly beneficial for firm performance in Pakistan. Bonazzi and Islam [140] discovered that agency cost can be minimized by appointing a quality auditor, which highlights every financial and non-financial aspect of a firm clearly and truly. Multiple researchers provided evidence to support these results [141,142]. Seventhly, our outcomes reported that firm performance and dividend payment have a positive association. In this support, Farrukh, et al. [143] found a positive linkage between dividend policy and firm performance in Pakistan. Various studies suggested the positive connection between dividend payment and firm performance [144,145].

On the other side, our results discovered that corporate social responsibility and firm performance are positively linked. For the Pakistani market, Javeed and Lefen [24] supported the role of CSR for firm performance. Furthermore, multiple scholars have provided evidence for the positive association between CSR and firm performance [48,144]. Stakeholder theory supported these results as CSR is an imperative means to improve shareholder wealth and motivate more stakeholders for social actions [15]. 

Most importantly, the results of our study confirmed that all internal corporate governance factors (CEO POWER, BI, BS, MO, OC, AQ) have positive effects on firm performance with the moderating role of CSR. CSR is a valuable tool that controls, monitors, and publishes internal corporate governance activities [9]. Reputation is a major thing for the long-run survival of the firms, therefore, internal corporate governance factors focus on the social activities for the improvement of the firm image in the market [54]. Theoretically, stakeholder theory supports these findings as the internal corporate governance uses effective ways for saving the interest of the firm stakeholders with the help of CSR [9,19]. CSR put pressure on the firm internal corporate governance to work for firm performance and firm positive image in the market. Pakistani market already has a lack of trust of stakeholders, therefore, CSR serves as a strong tool to boost the relationship between corporate governance and shareholders. 

## 6. Conclusions

Corporate social responsibility has great importance for the industrial sector and it may control the industrial negative environmental impacts. Therefore, the object of this study is to evaluate the relationship between internal corporate governance and firm performance. Before this study, no study has used all of these internal corporate governance factors together. Secondly, this study examines the role of corporate social responsibility for firm performance. Importantly, we used corporate social responsibility as a moderating factor to find out the reason for the positive, negative, or insignificant relationship between internal corporate governance factors and firm performance. For this investigation, this study selected 339 Pakistani firms from 2010 to 2019, which covers 10 years, and developed 3950 observations. Multiple sources have been used to collect the data such as the Pakistan Stock Exchange (PSX), the State Bank of Pakistan (SBP), the Securities and Exchange Commission of Pakistan (SECP), sustainability reports, and the companies’ annual reports from their respective websites. This study applied various statistical techniques, such as OLS, FE, 2SLS, GMM, and FGLS, and concluded various outcomes.

The empirical results demonstrated that firm performance can be enhanced with a powerful CEO. Moreover, board independence has a significant and positive association with firm performance. Board size has a significant and positive influence on firm performance. Besides, managerial ownership and firm performance also presented significant and positive relationships with each other. Furthermore, ownership concentration and firm performance have also significant and positive connections. Furthermore, this study’s results revealed that audit quality and firm performance have a significant and positive relationship. Besides, our findings discovered that CSR have a positive and significant relationship with firm performance.

Imperatively, this study applied corporate social responsibility as a moderating factor to inspect the association between internal corporate governance and firm performance. Our findings revealed that all internal corporate governance factors such as CEO power, BI, BS, OC, MO, and AQ positively influence firm performance with the moderating role of corporate social responsibility. Based on these results, we can suggest that the moderating role of corporate social responsibility plays an important role in the positive relationship between internal corporate governance and firm performance.

### 6.1. Policy Implications

In light of these outcomes, this study has numerous suggestions and policy implications for policymakers, owners, investors, shareholders, and governments of both developing and developed economies to improve corporate governance and social activities. The following are important implications of this study: First, firms should abide by the guidance of regulatory bodies for the improvement of corporate governance and social practices. Second, firms should identify and implement the proper actors of corporate governance for the improvement of firm performance. Third, the corporate governance main bodies such as CEOs, board members, and ownership structure have a superior role to follow and implement the corporate governance activities. While making corporate strategies, the present study sheds light on the important role of the executives, managers, ownership structure, regulatory bodies, and corporate social aspects to execute the strategies for the sake of shareholders’ interests. Furthermore, managers are warned of the reality that their actions are being watched and supervised by the top executives and committees, thus they are accountable for their activities.

Fourth, firms should also focus on corporate social responsibility for the improvement of firm performance and internal corporate governance practices. Fifth, the Government of Pakistan (GOP) should formulate strict regulations of corporate social practices that monitor the actions of internal corporate governance. This study suggests that Pakistani regulatory bodies, for focusing on corporate social responsibility, should control the internal corporate governance in the firms where family ownership and unstable political conditions exist. Sixth, the GOP should present awards and benefits to those firms that are properly publishing information about corporate governance and social practices. Importantly, the whole world is suffering from environmental issues that lead to global warming, and researchers believe that industrial sectors are major sources of those environmental problems. Thus, governments and regulatory authorities should focus on corporate social responsibility for firms, which may reduce those industrial negative impacts on the environment. Thus, the findings of this study are helpful for governments and policymakers to form stringent regulations that can ultimately improve the performance of industrial sectors. Firms of developing countries are the major source of environmental problems; thus, this study proposes that proper implementation of corporate social practices may reduce these problems.

### 6.2. Limitation and Future Research Directions

This study is limited to a single country for the investigation of the association between corporate governance and firm performance. Moreover, this study used data for 10 years because of data availability; therefore, this study period could be extended for future research. Because of data constraints, this study used few variables for internal and external firm performance. Additionally, the role of women is highly important for organizational performance and it is also the main agenda of corporate social aspects [146]. Therefore, the role of women on board is being enhanced for the improvement of profitability and firm social practices. Even, the United Nations (UN) 2030 plan also focuses on the role of women on board and firm top management [147]. 

Furthermore, for future research, this study can be extended by focusing on the role of women on the board and other corporate governance variables. Financing configuration and organization structure can be considered in future research. Besides, these governance variables can be used as a moderator in the future to prove their impact on firm performance. A study on corporate governance practices in multiple countries can also be conducted in the future.

## Figures and Tables

**Table 1:** Variables’ construction.

Dependent Variables	Abbreviations	Measures
Economic Value Added	EVA	Net operational profit after tax minus cost of capital into the capital invested [94].
Sustainable Growth Rate	SGR	PM × (1 − D) × (1 + L)/(T − (PM ×(1 − D) × (1 + L))) ^1^ [24].
**Independent Variables**		
Chief Executive Officer Power	CEO Power	CEO annual compensations/ Whole board of directors compensations [68].
Board Independence	BI	Independent directors on board/Total number of board of directors on board [95].
Board Size	BS	The total member of board members on the board [96].
Ownership Concentration	OC	The major shareholding portion or Top 5 shareholders [24].
Managerial Ownership	MO	The portion of shares held by the management [24].
Audit Quality	AQ	The statutory audit fees to the number of sales [97].
Corporate Social Responsibility	CSR	The addition of Earning Per Share (EPS), total taxes, staff salaries, interests, and public expenses minus social cost divided by total equity [24].
**Control Variables**		
Property, Plant, and Equipment	PPE	The ratio of property, plant, and equipment to total sales [71].
Firm Size	LNTA	The natural log of total assets [71].
Asset turnover	ATO	The ratio of total sales to total asset [71].
Environmental Awareness	EA	The ratio of an average green investment divided by No. of employees [71].

^1^ PM shows profit margin (existing and target), D indicates dividend payout ratio, L indicates target total debt to total equity ratio, and T indicates ratio of total assets to sales.

**Table 2:** Variables’ construction.

Variables	M	SD	1	2	3	4	5	6	7	8	9	10	11	12	13
1. EVA	0.27	0.5	1												
2. SGR	0.68	0.13	1.00 ***	1											
3. CEO POWER	3.38	4.21	0.78 ***	0.79 ***	1										
4. BI	2.43	5.01	0.98 ***	0.98 ***	0.77 ***	1									
5. BS	0.2	0.37	0.52 ***	0.53 ***	0.71 ***	0.50 ***	1								
6. MO	5.36	3.54	0.45 ***	0.44 ***	0.33 ***	0.47 ***	0.28 ***	1							
7. OC	1.24	1.64	0.73 ***	0.74 ***	0.92 ***	0.70 ***	0.66 ***	0.25 ***	1						
8. AQ	0.92	1.72	0.99 ***	0.97 ***	0.79 ***	0.99 ***	0.53 ***	0.46 ***	0.73 ***	1					
9. CSR	0.97	1.36	0.74 ***	0.73 ***	0.92 ***	0.72 ***	0.68 ***	0.30 ***	0.86 ***	0.73 ***	1				
10. ATO	−0.71	0.59	−0.44 ***	−0.63 ***	−0.32 ***	−0.46 ***	−0.17 ***	−0.43 ***	−0.27 ***	−0.47 ***	−0.20 ***	1			
11. EA	0.29	0.4	0.06 ***	0.02 ***	0.29 ***	0.09 ***	0.24 ***	0.42 ***	0.21 ***	0.07 ***	0.21 ***	−0.25 ***	1		
12. LNTA	0.11	0.13	0.25 ***	0.24 ***	0.29 ***	0.28 ***	0.22 ***	0.66 ***	0.24 ***	0.26 ***	0.18 ***	−0.49 ***	0.72 ***	1	
13. PPE	1.77	4.18	0.76 ***	0.75 ***	0.86 ***	0.79 ***	0.62 ***	0.53 ***	0.74 ***	0.78 ***	0.76 ***	−0.47 ***	0.45 ***	0.51 ***	1

Significance level *** *p* < 0.01.

**Table 3:** Endogeneity test.

Independent Variables	Model 1	Model 2	Model 3	Model 4	Model 5	Model 6	Model 7
CEO POWER	0.625 ***						
BI		1.288 ***					
BS			2.862 ***				
MO				0.051 **			
OC					0.261 ***		
AQ						1.423 **	
CSR							2.635 **
ATO							
EA							
LNTA							
PPE	10.827 **	6.429 ***	12.651 ***	7.956 **	13.733 **	9.617 ***	11.388 **
Constant	0.698 **	2.577 ***	0.737 ***	0.764 ***	1.492 **	0.468 ***	2.815 **
R^2^	0.8741	0.9216	0.8563	0.7889	0.9350	0.8819	0.8931
Durbin–Wu–Hausman	25.78 ***	31.57 ***	27.41 ***	28.74 ***	36.65 ***	33.44 ***	32.94 ***

Significance levels *** *p* < 0.01, ** *p* < 0.05.

**Table 4:** Results on ICG and FP.

Dependent Variables	Model 1		Model 2	
Independent Variables	EVA		SGR	
	FE	GMM	FE	GMM
CEO POWER	0.004 ***	0.005 ***	0.001 ***	0.011 ***
BI	0.015 ***	0.018 ***	0.003 ***	0.004 ***
BS	0.008 ***	0.004 **	0.002 ***	0.001 **
MO	0.006 ***	0.005 ***	0.001 ***	0.002 ***
OC	0.012 ***	0.009 ***	0.003 ***	0.003 ***
AQ	0.240 ***	0.237 ***	0.060 ***	0.059 ***
ATO	−0.004 ***	−0.005 **	−0.001	−0.002 **
EA	−0.007	−0.003	−0.015	−0.008
LNTA	−0.004	0.006	−0.002	−0.001
PPE	0.009 ***	−0.010 ***	−0.002 ***	−0.003 ***
Constant	−0.033 ***	−0.025 ***	−0.008 ***	−0.006 ***
R^2^	0.9920		0.9921	
F	31.54 ***		31.54 ***	
N	3686	2891	3686	2891
Hausman Test	58.86 ***		56.87 ***	
Wald Chi^2^		381,653.42 ***		382,654.44 ***

Significance levels *** *p* < 0.01, ** *p* < 0.05.

**Table 5:** Results on CSR and FP.

Dependent Variables	Model 3		Model 4	
Independent Variables	EVA		SGR	
	FE	GMM	FE	GMM
CSR	0.155 ***	0.144 ***	0.038 ***	0.035 ***
ATO	0.011	−0.009	0.002	−0.003
EA	−0.840 ***	−1.092 ***	−0.209 ***	−0.272 ***
LNTA	0.749 ***	0.655 ***	0.188 ***	0.164 ***
PPE	0.052 ***	0.054 ***	0.012 ***	0.013 ***
Constant	0.126 ***	0.229 ***	0.051 ***	0.058 ***
R^2^	0.6355		0.6412	
F	13.42 ***		13.54 ***	
N	3686	2891	3686	2891
Hausman Test	83.86 ***		84.85 ***	
Wald Chi^2^		4657.13 ***		4655.14 ***

Significance levels *** *p* < 0.01.

**Table 6:** Results of the impact of ICG on FP with the moderating role of CSR.

Dependent Variables	Model 5		Model 6	
Independent Variables	EVA		SGR	
	FE	GMM	FE	GMM
CEO POWER	0.002 ***	0.003 ***	0.006 ***	0.007 ***
BI	−0.008 ***	−0.007 ***	−0.002 ***	−0.003 ***
BS	0.004	0.005 **	0.001	0.004
MO	0.005 ***	0.004 ***	0.002 ***	0.001 ***
OC	0.008 ***	0.008 ***	0.001 ***	0.002 ***
AQ	0.050 ***	0.052 ***	0.012 ***	0.013 ***
CSR	−0.006 ***	−0.007 ***	−0.001 ***	−0.001 ***
CEO POWER × CSR	0.001 ***	0.002 **	0.009 ***	0.002 ***
BI × CSR	0.471 ***	0.524 ***	0.117 ***	0.131 ***
BS × CSR	0.856 ***	0.875 ***	0.214 ***	0.218 ***
MO × CSR	2.153 ***	1.976 ***	0.538 ***	0.494 ***
OC × CSR	1.935 ***	1.760 ***	0.483 ***	0.440 ***
AQ × CSR	10.88 ***	11.87 ***	2.722 ***	2.968 ***
ATO	−0.001	−0.003	-0.004	−0.007
EA	−0.002	−0.002 *	−0.006 ***	−0.009 ***
LNTA	−0.006 ***	−0.007 **	−0.001 **	−0.007 **
PPE	−0.001 ***	−0.001 ***	−0.003 ***	−0.004 ***
Constant	−0.008 **	−0.005	−0.002	−0.002
R^2^	0.9999		0.9998	
F	25.38 ***		26.37 ***	
N	3686	2891	3686	2891
Hausman Test	44.03 ***		45.44 ***	
Wald Chi^2^		29,303.31 ***		35,768.19 ***

Significance levels *** *p* < 0.01, ** *p* < 0.05, * *p* < 0.10.

**Table 7:** Robustness results of ICG and FP, CSR and FP, and ICG and FP with the moderating role of CSR.

Dependent Variables	Model 1	Model 2	Model 3	Model 4	Model 5	Model 6
Independent Variables	EVA	SGR	EVA	SGR	EVA	SGR
	FGLS	FGLS	FGLS	FGLS	FGLS	FGLS
CEO POWER	0.009 ***	0.002 ***			0.003 ***	0.008 ***
BI	0.007 ***	0.001 ***			−0.006 ***	−0.001 ***
BS	0.003 ***	0.002 ***			0.001	0.002
MO	0.002 **	0.0001 **			0.002 ***	0.004 ***
OC	0.001 ***	0.004 ***			−0.003 **	−0.005 **
AQ	0.264 ***	0.066 ***			0.036 ***	0.009 ***
CSR			0.141 ***	0.035 ***	−0.001 ***	−0.001 ***
CEO POWER × CSR					0.003 ***	0.004 ***
BI × CSR					0.304 ***	0.076 ***
BS × CSR					0.626 ***	0.156 ***
MO × CSR					2.761 ***	0.691 ***
OC × CSR					3.148 ***	0.787 ***
AQ × CSR					9.272 ***	2.318 ***
ATO	−0.002 ***	0.001 ***	−0.072 ***	−0.018 ***	−0.001 ***	−0.001 ***
EA	−0.005 ***	−0.012 ***	−0.356 ***	−0.090 ***	0.006 ***	0.002 ***
LNTA	−0.027 ***	0.006 ***	0.314 ***	0.078 ***	0.005 ***	−0.005 ***
PPE	0.011 ***	−0.002 ***	0.070 ***	0.017 ***	−0.001 ***	0.004 ***
Constant	−0.001 ***	−0.003 ***	0.025 ***	0.006 ***	−0.009 ***	−0.002 ***
N	3686	3686	3686	3686	3686	3686
Wald Chi^2^	972,427 ***	98,403.85 ***	52,988.46 ***	21,598.47 ***	63,849.06 ***	224,869.16 ***

Significance levels *** *p* < 0.01, ** *p* < 0.05.

## Data Availability

Not applicable.
